# Expression of TIP-1 Confers Radioresistance of Malignant Glioma Cells

**DOI:** 10.1371/journal.pone.0045402

**Published:** 2012-09-17

**Authors:** Miaojun Han, Hailun Wang, Hua-Tang Zhang, Zhaozhong Han

**Affiliations:** 1 Department of Radiation Oncology, Vanderbilt University, Nashville, Tennessee, United States of America; 2 Cancer Biology, Vanderbilt University, Nashville, Tennessee, United States of America; 3 Key Laboratory of Animal Models and Human Disease Mechanisms of the Chinese Academy of Sciences & Yunnan Province, Kunming Institute of Zoology, Kunming, Yunnan Province, China; 4 Graduate School, Chinese Academy of Sciences, Beijing, China; 5 Vanderbilt-Ingram Cancer Center, School of Medicine, Vanderbilt University, Nashville, Tennessee, United States of America; University of Nebraska Medical Center, United States of America

## Abstract

**Background:**

Malignant gliomas represent one group of tumors that poorly respond to ionizing radiation (IR) alone or combined with chemotherapeutic agents because of the intrinsic or acquired resistance. In this study, TIP-1 was identified as one novel protein that confers resistance of glioma cells to IR.

**Methodology/Principal Findings:**

Meta-analysis indicated that high TIP-1 expression levels correlate with the poor prognosis of human malignant gliomas after radiotherapy. Studies with established human glioma cell lines demonstrated that TIP-1 depletion with specific shRNAs sensitized the cells to IR, whereas an ectopic expression of TIP-1 protected the glioma cells from the IR-induced DNA damage and cell death. Biochemical studies indicated that TIP-1 protein promoted p53 ubiquitination and resulted in a reduced p53 protein level. Furthermore, p53 and its ubiquitination are required for the TIP-1 regulated cellular response to IR. A yeast two-hybrid screening identified that TIP-1, through its single PDZ domain, binds to the carboxyl terminus of LZAP that has been studied as one tumor suppressor functioning through ARF binding and p53 activation. It was revealed that the presence of TIP-1 enhances the protein association between LZAP and ARF and modulates the functionality of ARF/HDM2 toward multi-ubiquitination of p53, while depleting TIP-1 rescued p53 from polyubiquitination and degradation in the irradiated glioma cells. Studies with a mouse xenograft model indicated that depleting TIP-1 within D54 cells improved the tumor growth control with IR.

**Conclusions/Significance:**

This study provided the first evidence showing that TIP-1 modulates p53 protein stability and is involved in the radioresistance of malignant gliomas, suggesting that antagonizing TIP-1 might be one novel approach to sensitize malignant gliomas to radiotherapy.

## Introduction

Malignant gliomas are the most common malignant primary tumors arising in the human brain, treating these life-threatening tumors is still a clinical challenge. Although most of the glioma patients are treated with radiotherapy alone or combined with surgical resection and/or chemotherapies, outcomes of the treatment courses are still unsatisfactory partially due to either intrinsic or acquired tumor resistance to the cytotoxic treatment [1], [2].

The tumor suppressor p53 protein plays a vital role in the cellular responses to cytotoxic treatments through regulating cell cycle progression, DNA damage repair, and cell death. Upon stimulation of intrinsic or extrinsic factors, p53 protein is activated to sense, transduce and execute cellular responses. Human double minute 2 (HDM2) (or the murine homologue, MDM2 in mouse) has been well characterized as a negative regulator of p53 through two main mechanisms: direct binding to the N-terminal end of p53 protein and inhibiting p53-regulated transcription [3], [4], or targeting p53 for ubiquitination and subsequent degradation through the 26S proteasome [5]. HDM2, in turn, is regulated by p14/p19 ARF (alternative reading frame) which binds to HDM2 and limits the HDM2-regulated p53 inhibition [6]. Dysfunctional mutations in the ARF/HDM2/p53 pathway, such as over activation of HDM2 or a loss-of-function mutation of p53, have been demonstrated as mechanisms by which tumor cells acquire the unlimited proliferation potentials and resistance to apoptosis in a broad spectrum of tumors [7], [8]. In fact, restoring the functionality of p53 protein has been proposed and tested for clinical treatment of cancers [7], [9]. However, in spite of the fact that a significant portion of the primary malignant gliomas express the wild-type p53 protein, the status of p53 expression has a poor prognostic value in predicting the therapeutic outcomes of the primary malignant gliomas [10], [11]. Therefore, more studies are needed to understand the complexity of p53 activation and functionality in tumor progression and response to therapeutics. Recently, LZAP (also known as CDK5 regulatory subunit-associated protein 3 or C53) has been characterized as a tumor suppressor that physically associates with ARF and activates p53 in head-and-neck carcinoma cells [12]. It was found that LZAP regulates the susceptibility of breast cancer cells to genotoxic stresses including IR and several chemotherapeutic agents [13]. However, a recent report [14] has documented a controversial function of LZAP in hepatocellular carcinomas suggesting the complexity of its functionality under influence of cellular context in different type of tumors.

TIP-1 was firstly identified through yeast two-hybrid screening as one protein interacting with HTLV-1's tax protein [15]. After that, several more interacting partners and its biological functions in cell proliferation, stress response, development and cell polarization have been reported [16]–[22]. TIP-1 is conservative among species [18]. Compared to other PDZ proteins that usually contain multiple structural domains and thus function as scaffold proteins in the assembly of macromolecular machineries [23], [24], TIP-1 protein is primarily composed of a single PDZ (PSD-95/DlgA/ZO-1) domain. The unique structural identity suggests that TIP-1 might function differently from other PDZ proteins. TIP-1 demonstrated inhibitory activity on the beta-catenin-driven gene expression and proliferation of colon cancer cells [17]. It mediates the serum response through interacting with Rhotekin [20], it was identified as one essential components for the HPV-16 E6 oncoprotein-driven cell transformation [19]. We have characterized the translocation of the predominantly intracellular TIP-1 protein onto the cell plasma membrane of tumor cells as one biomarker of tumor response to ionizing radiation (IR) before the onset of apoptosis or cell death [25]. Elevated expression of TIP-1 has been found in human invasive breast cancers and associated with pulmonary metastasis in mouse models [26]. However, the biological functions, particularly in oncology, of this unique PDZ domain protein are elusively unclear.

In this report, we further studied the roles of TIP-1 in the IR response of malignant gliomas. Meta-analysis indicated that high levels of TIP-1 expression are associated with the poor prognosis of human malignant gliomas after radiotherapy. Studies with established cell lines *in vitro* and *in vivo* revealed a novel function of TIP-1 in the cellular response of human malignant gliomas to IR, and provided the first evidences suggesting that antagonizing TIP-1 might represent one novel approach to sensitize malignant gliomas to radiotherapy.

## Materials and Methods

### Cell culture, antibodies, and chemicals

Human glioblatoma cell lines (D54, U87 and T98G) were obtained from Dr. Yancie Gillespie (University of Alabama-Birmingham, Birmingham, AL) and purchased from American Type Culture Collection (ATCC, Rockville, MD, USA), respectively. The cells were maintained in DMEM/F12 medium supplemented with 10% fetal calf serum, 1% sodium pyruvate, and 1% penicillin/streptomycin (Thermo Scientific Inc., Waltham, MA). Cells were irradiated with 300 kV X-rays using a Pantak Therapax DXT 300 X- ray unit (Pantak, East Haven, CT). Rabbit anti-human TIP-1 antibody was produced as described previously [25]. Antibodies against LZAP, HDM2, DNA-PK_CS_, p53, and Ub(P4D1) were purchased from Santa Cruz Biotechnology (Santa Cruz, CA). Antibodies recognizing the cleaved caspase-3, phospho-Chk1 (Ser317), phospho-Chk2 (Thr68), and phospho-Histone H3 (Ser10) were purchased from Cell Signaling Technology (Danvers, MA). Antibodies against actin and ARF, and HRP- or fluorescence-labeled secondary antibodies were obtained from Invitrogen (Carlsbad, CA). Nutlin-3 (one HDM-2 inhibitor) was purchased from Sigma and dissolved in DMSO as stock. Other chemicals were purchased from Sigma (St. Louis, MO) unless otherwise stated.

### Recombinant plasmids, mutagenesis, and protein production

Recombinant pcDNA3.1 plasmids expressing cMyc-tagged TIP-1 protein, including the wild type (WT) or a mutant (MUT) with a dysfunctional PDZ domain (H90A) [16], were kindly provided by Dr. Paul A. Welling at University of Maryland (Baltimore, MD). A recombinant plasmid encoding FLAG-tagged LZAP [12] was a generous gift from Dr. Wendell G. Yarbrough at Vanderbilt University (Nashville, TN). QuikChange™ Site-Directed Mutagenesis Kit (Stratagene, Santa Cruz, CA) was used to create a LZAP mutant at a putative C-terminal PDZ-binding motif (from -LMGTSL to-LMGASA, the mutations are underlined) with primers 5′-CGGAAGCTTGGTACCATGGAG-3′ and 5′-CCGTCTAGATCAGGCAGAGGCTCCCATCAGGTTCAC-3′ (Sigma). All the constructs were confirmed with DNA sequencing. GST or GST-tagged TIP-1 proteins were expressed in *E. coli* and purified to homogeneity as described [25].

### RNA interference and cell transfection

Constructs expressing small hairpin RNA (shRNA) were purchased from Open Biosystems (Huntsville, AL). Two TIP-1 specific shRNAs (TIP-1 #1: 5′-GGCTAACAGCTGATCCCAA-3′ and TIP-1 #2: 5′-GCAAAGAGTTGAAATTCACAA-3′) match with different region of human and mouse TIP-1 mRNA transcripts. A validated shRNA control with no obvious matched sequence in human and mouse genome was also used. Small Interfering RNA (siRNA) for the LZAP knockdown were designed according to the published data [13] and synthesized by Sigma. Three LZAP siRNAs used in this study include CS3-1: r(GCAGAUUGCCAAGUGCCAGC), CS3-2: r(GCAGGAGAUUAUAGCUCUGUA), and CS3-3: r(GAGGCCUCCACGAAGAAUAUU). A control siRNA was purchased from Qiagen (Germantown, MD). Interfering RNA pool was used for silence p53 (siRNA-1: GGAGAAUAUUUCACCCUUC; siRNA-2: GCAGUCAGAUCCUAGCGUC; siRNA-3: GUGCAGCUGUGGGUUGAUU; and siRNA-4: GAAAUUUGCGUGUGGAGUA. Thermo Scientific Inc., Waltham, MA). RNAiMAX (Invitrogen) was used for the siRNA transfection by following the manufacturer's instructions. Transfection of cells with the recombinant plasmids and the establishment of stable clones were conducted with standard protocols [27]. The protein expression level was detected by western blot analysis of whole cell lysate with the specific antibodies.

### Clonogenic assay and detection of apoptosis

Cells were disaggregated, counted with hemocytometer, plated on new plates with fresh medium and allowed to attach for around 5 hours. The cells were then irradiated at 0 (mock treatment), and variable doses of X-ray, respectively. After 10 to 14 days, colonies were counted after staining with 1% methylene blue. Cell survival fractions were calculated to determine dose modification factor (DMF) upon the dose ratio at the survival fraction of 0.1 [25]. To detect cell apoptosis, the cells exposed to the mock treatment or 10 Gy X-ray were collected at 24 or 48 hours post treatment. Cell apoptosis was detected by western blot analyses of the cleaved caspase-3 within cell lysates. The blots were scanned using a densitometer to quantify the proteins of interest. The semi-quantitative measurements were sequentially normalized to actin controls and those from the cells transfected with a respective vector control (counted as 1). Cells were also stained with Annexin V and propidium iodide (PI) (BD pharmingen, San Diego, CA) and analyzed with flow cytometry [25].

### γ-H2AX foci staining

Cells cultured on sterile glass slides were exposed to a mock treatment or 5 Gy X-ray. After the specific time points after the treatment, the cells were fixed and stained with mouse anti–phospho-γ-H2AX antibody (Abcam, 1∶500), followed by anti-mouse Alexa Fluor 488–conjugated antibody (Invitrogen, 1∶2000), or anti-mouse Alexa Fluor 594–conjugated antibody (Invitrogen, 1∶2000). DAPI (4′,6-diamidino-2-phenylindole) staining was used to visualize the cell nucleus. The images were acquired by using a fluorescent microscope (×400; Carl Zeiss). Quantification of the γ-H2AX foci was conducted as previously described [28], as cells containing more than 5 foci were counted as positive. At least 100 cells from each of the triplicate experiments were counted.

### Comet assay

Cells were exposed to 5 Gy X-ray or a mock treatment. After 10 minutes, 4 and 24 hours post irradiation, the cells were collected and combined with low melting agarose before being applied onto CometSlides for electrophoresis according to the manufacturer's instructions (Cell biolabs, San Diego, CA). Cells were then visualized using fluorescent microscopy (Carl Zeiss). At least 75 comet images were obtained for each time point and analyzed using Comet Score software (Comet Assay IV). Percentages of cells with comet tail as well as comet Tail Moment were calculated upon more than 100 cells from each of three independent experiments [29].

### Yeast two-hybrid screening

TIP-1 interacting proteins were identified through a yeast two-hybrid screening of human fetal brain cDNA libraries. Screening, hits validation, and identification were performed at the Duke University yeast two-hybrid screening facility (Durham, NC).

### Pull-down and co-immunoprecipitation

Cells were harvested and resuspended in lysis buffer (50 mM Tris-HCl, pH 7.2, 150 mM NaCl, 5 mM EDTA, 1% Nonidet P-40 supplemented with protease inhibitor cocktails and phosphatase inhibitor cocktail 1 and 2). After the cell debris were removed, 50 µg of the purified protein (GST, GST-tagged wild type or mutant TIP-1) were incubated with the cell lysates for 2 hours at 4°C, followed by incubation with 75 µl GST-binding resins (Novagen, Gibbstown, NJ) for another 2 hours, respectively. After two washes with the lysis buffer, the resin-associated proteins were eluted with 10 mM glycine (pH 2.0). The samples were then subjected to SDS-PAGE and immunoblot analyses. In the immunoprecipitation assays, 500 µg of cell lysate and 2 µg of antibody were incubated with 100 µl of Protein A plus G agrose slurry (Santa Cruz Biotech) at 4°C for 2 hours, the co-immunoprecipitated proteins were analyzed with specific antibodies after SDS-PAGE fractionation.

### p53 ubiquitination assay

p53 ubuquitination was detected as previously described [30]. Briefly, at 8 hours post irradiation (5 Gy or mock treated), the cells were incubated with 25 µM proteasome inhibitor MG132 (Sigma) for 4 more hours before the cells were suspended in a lysis buffer (50 mM Tris-HCl, pH 7.2, 150 mM NaCl, 5 mM EDTA, 1% Nonidet P-40 supplemented with protease inhibitor cocktails). p53 was immunoprecipitaed from the cell lysate with a rabbit anti-p53 antibody (FL393) for SDS-PAGE and immunoblot analysis with a mouse antibody against ubiquitine.

### Tumor growth studies

One million of the disaggregated tumor cells within 50 µL of phosphate buffered saline (PBS) were subcutaneously inoculated in the both hind limbs of FoxN1-null nude mice (Harlan Laboratories, Prattville, AL). Tumor size was measured with caliper in every other days and calculated as described [31]. When tumor reached 0.3 cm in diameter, X-ray radiation was delivered through a Pantak Therapax DXT 300 X- ray unit (Pantak, East Haven, CT) at 3 Gy for five consecutive days. Only the tumors in the right hind limbs were irradiated while the tumors in the left hind limbs of the same animals were used as mock radiation treatment control. Animals were sacrificed when tumor size was bigger than 1 cm in diameter or tumor growth affected the mouse behaviors or motility. All the animal studies were conducted as approved by the Institutional Animal Care and Use Committee (IACUC) at Vanderbilt University.

### Statistical analyses

Microarray data for human glioblastoma multiformes were from “The Cancer Genome Atalas” project [32]. Patients with elevated TIP-1 expression levels (log2 tumor/normal ratio >1.5) are defined as “TIP-1 high” group, those with the ratio less than 0.5 are defined as “TIP-1 low” group. Survival curves were plotted with the Kaplan-Meier method and compared with a log-rank test. The Student's *t* test was used to determine the statistical significance unless otherwise stated. All quantitative data was presented as mean +/− standard deviation.

## Results

### Elevated TIP-1 expression levels correlate with the poor prognosis of human malignant gliomas after radiotherapy

**Figure 1:**
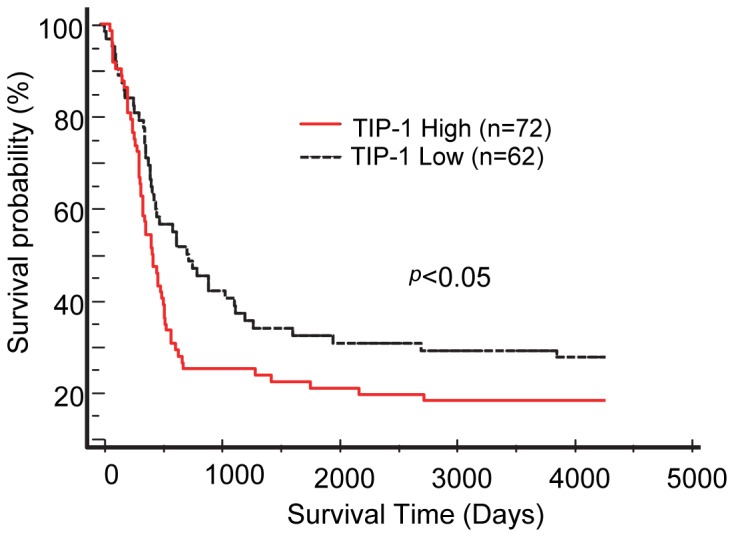
TIP-1 expression levels correlate with the survival probability of patients with gliobmatoma multiforme. Patients with elevated TIP-1 expression levels (log2 tumor/normal ratio >1.5) are defined as “TIP-1 high” group, those with the ratio less than 0.5 are defined as “TIP-1 low” group. Kaplan-Meier survival probability curves were developed with MedCalc. Statistical analysis was conducted with Log Rank Test (*p*<0.05).

To determine the prognostic value of the TIP-1 expression in glioma patients after radiotherapy, we analyzed the microarray datasets from “The Cancer Genome Atalas” project [32]. All these brain tumors are glioblastoma multiformes (GBM, grade IV according WHO classification) and express wild-type TIP-1. Patients with elevated TIP-1 expression levels (log2 tumor/normal ratio >1.5) are defined as “TIP-1 high” group, those with the log2 tumor/normal ratio less than 0.5 are defined as “TIP-1 low” group. Kaplan-Meier survival curves were developed for those patients (Fig. *1*). Statistical analysis using Log Rank Test indicated that TIP-1 expression levels correlate to the survival probability of GBM patients after radiotherapy (*p*<0.05), the patients with elevated TIP-1 expression levels experienced a poor prognosis after radiotherapy.

### TIP-1 knockdown sensitizes glioma cells to X-ray radiation

**Figure 2:**
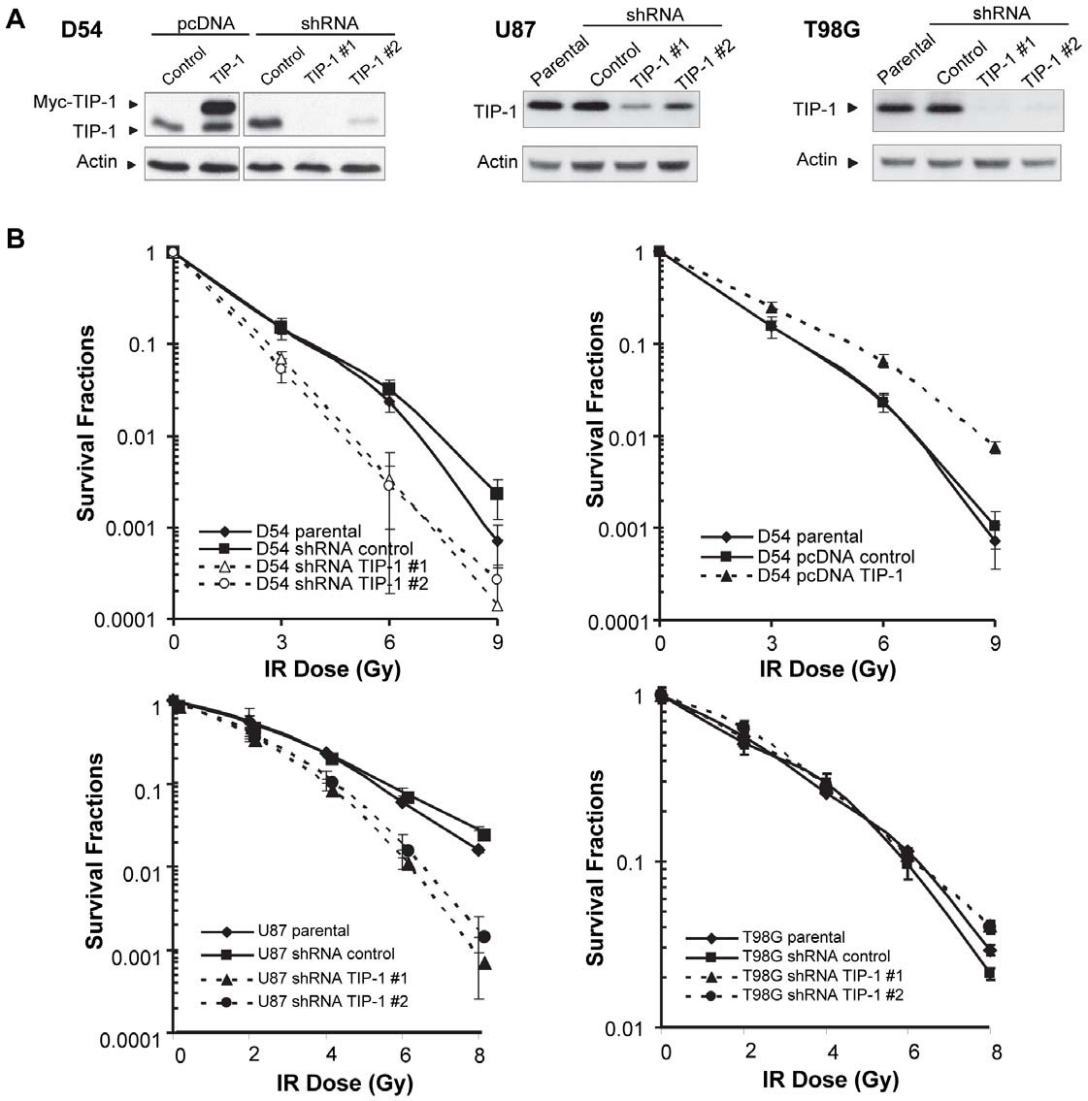
TIP-1 knockdown sensitizes glioma cells to ionizing radiation. *A*) Western blot analyses of TIP-1 expression in D54, U87 and T98G cells in which TIP-1 protein expression was genetically manipulated with recombinant plasmids or shRNA respectively. Two independent shRNA sequences were used for TIP-1 knockdown. *B)* Survival fractions of D54, U87 and T98G cells after single dose X-ray radiation treatment were determined with clonogenic assays. Vector control and parental cells were included in the assays. Shown are representative data from at least three independent experiments performed in triplicate.

The putative roles of TIP-1 expression in the cellular response of glioma cells to ionizing radiation were studied with clonogenic assays. Stable cell lines with ectopic TIP-1 expression or shRNA downregulation were established, and the TIP-1 protein expression in those cells was verified by western blot analyses of the cell lysates with specific antibodies (Figs. *2A*). Two independent shRNA sequences for TIP-1 knockdown were used to prevent bias from off-target effects. In the clonogenic assays, the ectopic expression or depletion of TIP-1 did not affect the cell plating efficiency (data not shown). Compared to the vector control or parental cells, TIP-1 knockdown with shRNA sensitized both of D54 and U87 cells to IR, but had no significant impact on T98G cells (Fig. *2B*). A dose modification factor (DMF) of 1.54 +/− 0.10 and 1.23 +/− 0.07 were obtained at the 0.10 survival fraction for TIP-1 knockdown in D54 and U87 cells, respectively. TIP-1 ectopic expression had a dose-modification factor as of 0.71 +/− 0.12 in D54 cells.

### TIP-1 expression levels correlate with the apoptosis rate of IR-treated D54 cells

**Figure 3:**
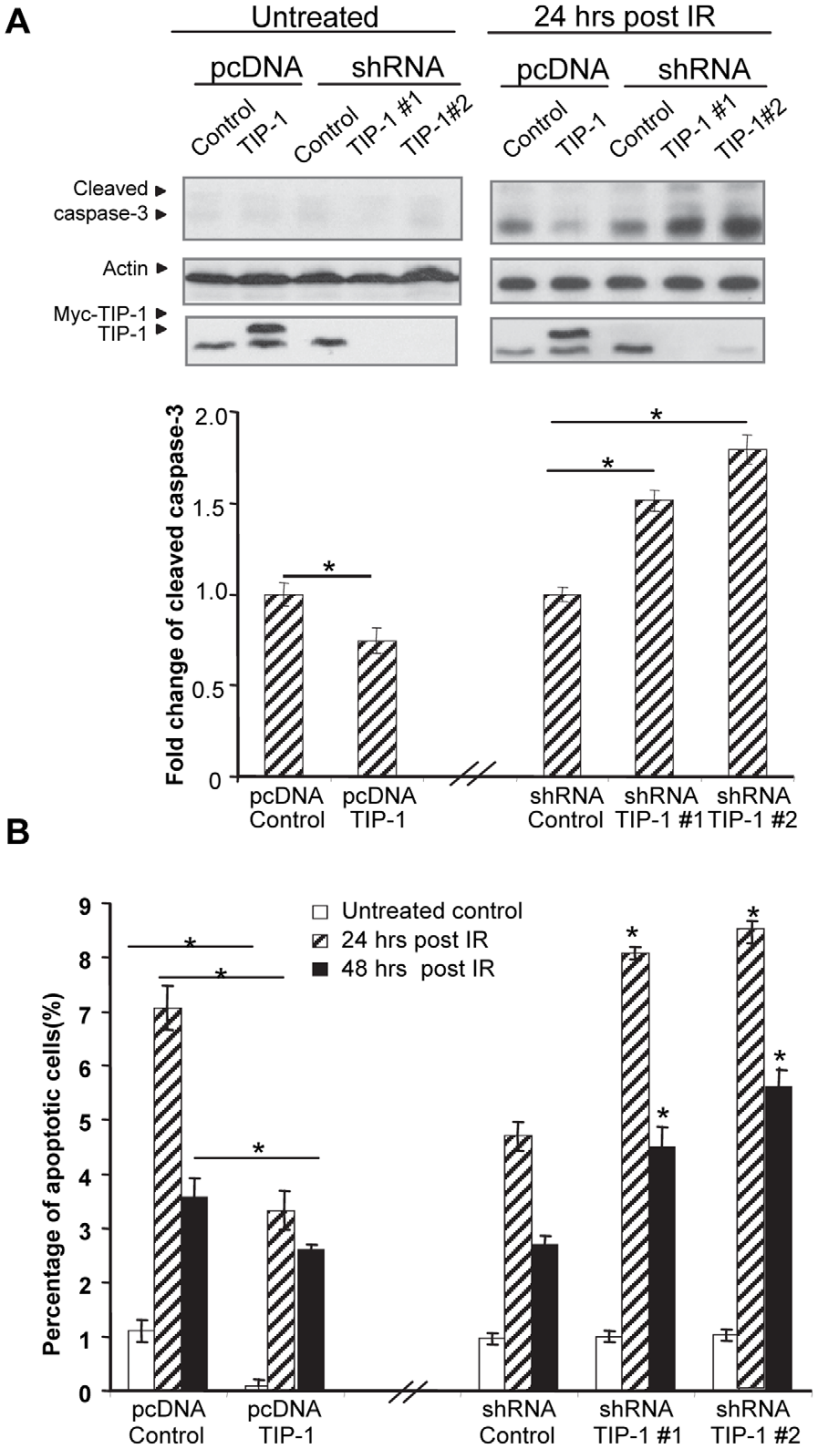
TIP-1 protein levels correlate to the radiation-induced apoptosis in D54 cells. *A)* Caspase-3 cleavage within D54 cells were examined with western blot. Cells received mock (untreated) or 5 Gy IR treatment were analyzed 24 hours post the treatment. The blots were scanned using a densitometer to quantify the proteins of interest. Relative fold change of caspase-3 activation for each cell line was normalized to the respective vector control (counted as 1). *B)* Flow cytometric analyses of Annexin V/PI stained cells. Cells were analyzed at the indicated time points post the mock or 10 Gy IR treatment. Shown are mean +/− standard deviation of at least three independent experiments performed in triplicate. Significance was determined by comparing the denoted group to the respective vector control with the same treatment, respectively. * *p*<0.05 with t-test.

Apoptosis was detected with western blot analysis of caspase-3 cleavage and flow cytometric profiling of PI- and Annexin V-stained cells. Semi-quantitative measurements were used to calculate fold changes of the caspase-3 activation before and after IR treatment, the fold changes were normalized to the respective vector control (counted as 1). Even though D54 cells are extremely resistant to IR-induced apoptosis, TIP-1 knockdown caused a prominent increase in the caspase-3 cleavage, whereas an ectopic expression of TIP-1 was associated with a reduced caspase-3 cleavage at 24 hours post a single dose of 5 Gy irradiation in those cells (Fig. *3A*). Flow cytometric profiles of cell apoptosis demonstrated a similar trend. At 24 hours post a single dose of 10 Gy irradiation, few (∼3.3%) of D54 cells with an ectopic expression of TIP-1 underwent apoptosis compared to around 7% cell apoptosis for the vector control, while TIP-1 knockdown caused around 8.4% apoptosis compared to less than 5% cell apoptosis for cells with a control shRNA (Fig. *3B*). This trend was extended to 48 hours post the irradiation in D54 cells.

### TIP-1 facilitates DNA damage repair after IR treatment within glioma cells

**Figure 4:**
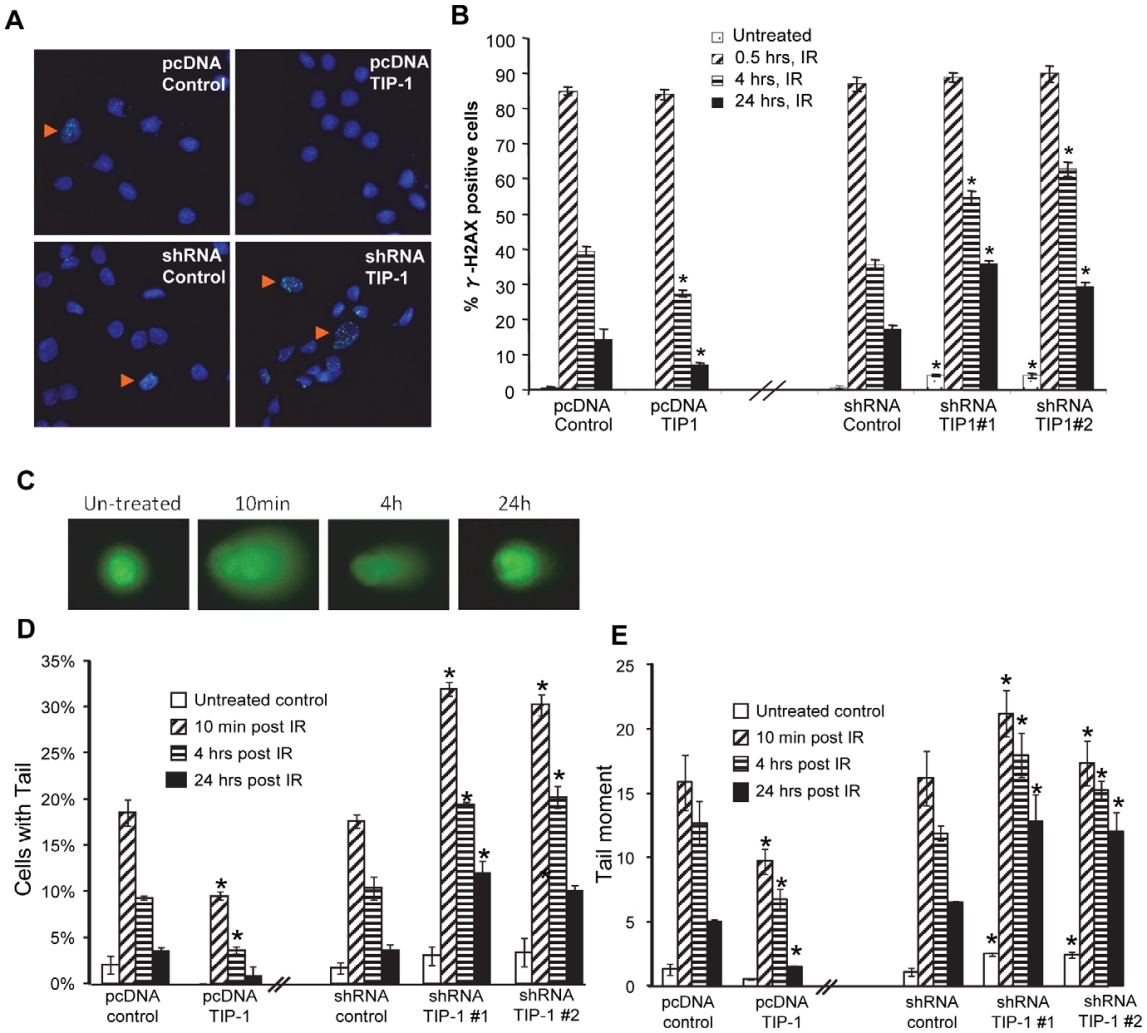
TIP-1 facilitates DNA damage repair after IR treatment. *A*) γ-H2AX foci staining. D54 cells were mock treated or irradiated at 5 Gy, and γ-H2AX foci were detected by immunofluorescence staining at 0.5, 4 and 24 hours post the treatment. The representative images show positive cells (pointed with arrow heads) containing more than 5 foci. *B)* Percentage of γ-H2AX foci-positive cells was calculated upon at least 100 cells from each of the triplicate experiments. *C)* Representative images (×400) of IR-induced DNA damage visualized with neutral comet assay. *D)* Percentage of cells with comet tails (as one indicator of popularity of DNA strand breaks among cells). *E)* Statistics of tail moments (as one indicator of DNA damage severity in individual cells). At least 75 comet images (including more than 100 cells) were analyzed for each group. Shown are the mean +/− standard deviation of at least three independent experiments. Significance was determined by comparing the denoted group to the respective vector control with the same treatment, respectively. * *p*<0.05, t-test.

DNA damage were detected with γ-H2AX foci staining and comet assays [29]. In the γ-H2AX foci staining assays, only the cells with more than 5 foci were counted as positives (Fig. 4*A*). The quantitative analyses (Fig. *4B*) showed that the majority (∼85%) of D54 cells contained IR-induced γ-H2AX foci at 30 minutes after 5 Gy irradiation. Different ratio of γ-H2AX foci-positives in the cells with various levels of TIP-1 expression were observed at 4 hours and even more remarkable at 24 hours post the IR treatment. Compared to the vector control which had around 15.1% of γ-H2AX foci-positive cells at 24 hours post 5Gy IR, D54 cells with an ectopic expression of TIP-1 had less (around 6.99%) of γ-H2AX foci-positive cells at the same time point after such IR treatment. However, D54 cells with TIP-1 knockdown had around 29.5% to 35.8% γ-H2AX foci-positives compared to the vector control which had only around 20% γ-H2AX foci-positives at 24 hours post 5Gy IR We also observed a remarkable portion of the cells with TIP-1 knockdown were stained as γ-H2AX foci positive even without IR treatment (Figs. *4B*), suggesting a potential role of TIP-1 in maintaining the genomic integrity within glioma cells.

Such impact of the TIP-1 expression on IR-induced DNA damage was also observed in neutral comet assays, in which DNA double strand-breaks leads to a “tail-like” morphology of cell nucleus after electrophoresis (Fig. 4*C*). Cells with such tail were counted to quantify the DNA damage in the cell population, while tail length (Tail Moment) of individual cells was measured to demonstrate the severity of the DNA damage in individual cells [33]. Compared to D54 cells transfected with a vector control that had ∼18% tail-positive cells at 10 minutes after a single dose of 5 Gy irradiation, D54 cells with an ectopic TIP-1 expression were only 10% tail-positive under same condition (Fig. 4*D*). Similarly but more remarkably, TIP-1 knockdown increased the tail-positive cells from ∼18% (shRNA control) to 31-34% (Fig. *4D*) at 10 minutes after 5 Gy irradiation. Consistent to the γ-H2AX foci staining, tail moment analyses (Fig. 4*E*) indicated that the cells with TIP-1 depletion experienced more severe genome integrity, even without IR treatment.

### TIP-1 suppresses p53 activation in the irradiated glioma cells

**Figure 5:**
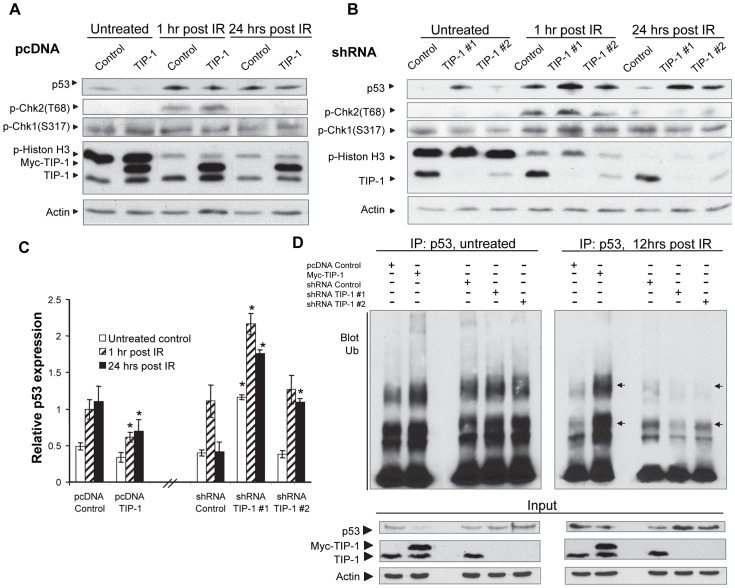
TIP-1 suppressed p53 activation after irradiation. D54 cells with an ectopic expression *(A)* or shRNA depletion *(B)* were mock treated or irradiated at 5 Gy, whole cell lystates were collected at the indicated time points post the treatment for western blot analyses to profile pathway mediators that sense, transduce, or execute cellular response to DNA damage. *C)* Semi-quantification of p53 upon the band density on blots using a densitometer. Significance was determined by comparing the denoted group to the respective vector control with the same treatment, respectively. *p<0.05, t-test. *D)* Analyses of p53 ubiquitination. Cells were mock treated or with IR (5 Gy). MG132 was added at 8 hours post the treatment and incubated for 4 more hours before the cell lysates were collected to analyze the p53 ubiquitination. The ubiquitinated p53 protein was immunoprecipitated with antibody against p53 and blotted with ubiquitin-antibody after the proteins were separated with SDS-PAGE. The representative image shows the ubquitinated p53 ladder (pointed with arrows), as well as overall p53, TIP-1 and actin in each cell lysate.

Western blot analyses were applied to determine the pathway mediators that sense, transduce, or execute the cellular responses to the IR-induced DNA damage. We detected overall protein levels of p53, the phosphorylation status of Chk1 (Ser317), Chk2 (Thr68) and Histone H-3 (Ser10) within the whole D54 cell lysates before and after IR treatment. At the indicated time points, modulating the TIP-1 protein level had a minor impact on the phosphorylation status of histone-3, Chk1 or Chk2 (Figs. *5A, B*). Interestingly, we found that an ectoptic expression of TIP-1 inhibited the p53 protein accumulation after IR, while the TIP-1 knockdown was associated with an elevated p53 protein accumulation after irradiation (Figs. *5A–C*). With the presence of a proteasome inhibitor MG132, western blot analysis of p53 ubiquitination status further revealed that an ectopic expression of TIP-1 promoted the polyubiquitination of p53 protein, while the TIP-1 depletion markedly reduced the p53 protein polyubiquitination within the irradiated D54 cells (Fig. *5D*). These observations suggested that p53 might mediate the TIP-1-regulated susceptibility of glioma cells to IR. This hypothesis is also support by the results from clonogenic assays (Fig. *2B* *)*, in which manipulating TIP-1 expression had a minor impact on T98G cells which express a p53 mutant, but had dramatic influence on the radiosensitivity of D54 and U87 cells with wild-type p53 protein.

### p53 mediates the TIP-1-regulated cellular response to IR of glioma cells

**Figure 6:**
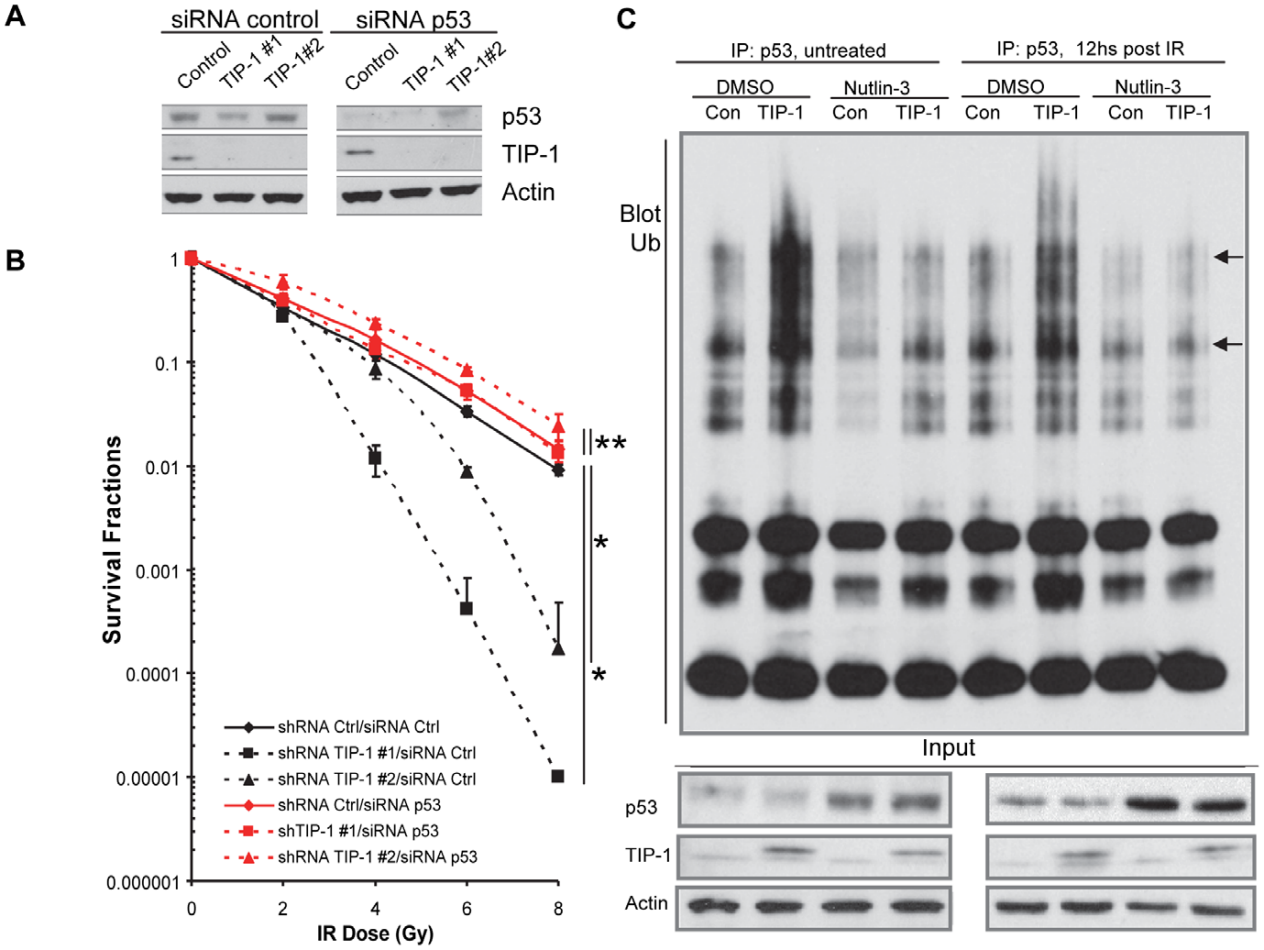
p53 mediates the TIP-1-regulated cellular response to IR of glioma cells. *A)* Western blot analysis of p53 expression within D54 cells with variable TIP-1 expression levels that were transfected with a control or p53-targeted siRNAs, respectively. Cell lystates were analyzed 72 hours post the siRNA transfection. *B)* Cell survival fractions in clonogenic assays after radiation treatment at variable doses (0, 2, 4, 6, and 8 Gy). Shown are the representative data of at least three independent experiments performed in triplicate. Significance was determined by comparing the denoted group to the respective vector control. * *p*<0.05, ** *p*>0.05, all tests of significance were determined with a two-sided Student's *t* test. *C)* p53 ubiquitination status with or without HDM2 inhibitor (Nutlin-3). D54 cells with or without TIP-1 ectopic expression were incubated with DMSO (vehicle control) or Nutlin-3 (10 µM in DMSO) for 2 hours before the culture was mock treated or with IR (5 Gy). MG132 was added at 8 hours post the IR treatment and incubated for 4 more hours before the cell lysates were collected to analyze the p53 ubiquitination as described. The representative image shows the ubquitinated p53 ladder (pointed with arrows), as well as overall p53, TIP-1 and actin in each cell lysate.

The putative roles of p53 and its ubiquitination in the TIP-1 regulated radioresistance of glioma cells were studied with D54 cells. A pool of validated small interference RNAs (siRNAs) were selected to silence p53 expression in D54 cells with different TIP-1 expression levels (Fig. *6A*). Clonogenic assays indicated that p53 knockdown attenuated the biological impact of TIP-1 expression on the radiosensitivity of D54 cells (Fig. *6B*). Evaluations based upon dose modification factors or two-sided Student's *t*-test of the data sets indicated that, after p53 knockdown, D54 cells demonstrated radiosensitivity independent of the TIP-1 expression levels. This data suggests that TIP-1 regulated radioresistance of glioma cells, at least in part, through p53. Furthermore, inhibition of HMD-2 with nutlin-3 partially eliminated the effects of TIP-1 expression on the p53 polyubiquitination and protein accumulation in the irradiated D54 cells (Fig. *6C*), indicating that HDM-2 is involved in the TIP-1 regulated p53 inhibition in glioma cells.

### TIP-1 physically interacts with LZAP within glioma cells

**Figure 7:**
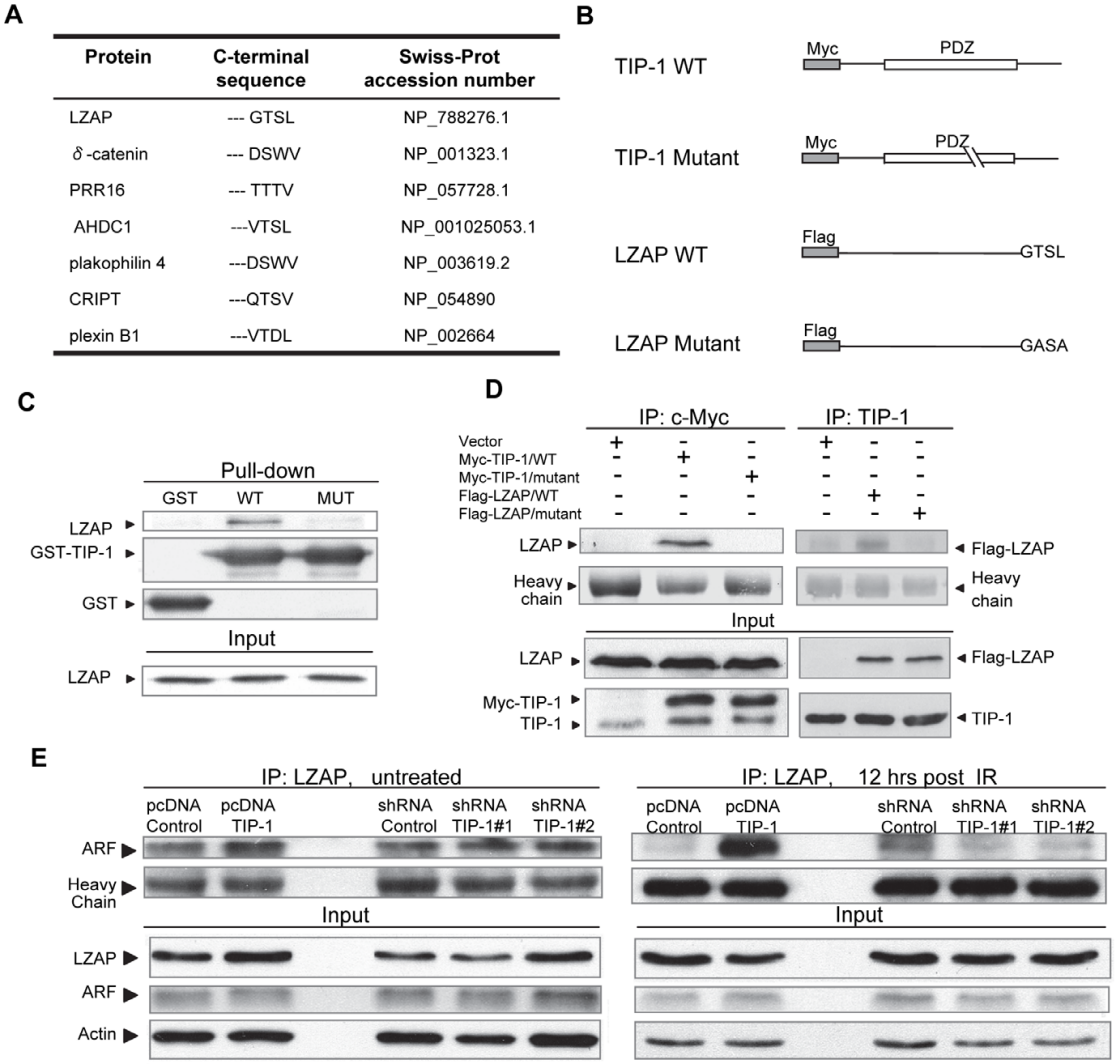
TIP-1 interacts with LZAP and enhances the protein interaction between LZAP and ARF within the irradiated D54 glioma cells. *A)* Putative TIP-1 interacting proteins identified through yeast two-hybrid screening of a human fetal brain cDNA library. *B)* Schematic illustration of the constructs used for studies of the protein interaction between TIP-1 and LZAP. The specific interaction of TIP-1 and LZAP was validated with pull down assay *(C)* with recombinant GST-fused TIP-1 (WT) protein or a mutant (MUT) with a dysfunctional PDZ domain, as well as co-immunoprecipitation assay *(D)* by using D54 cells that were co-transfected with recombinant plasmids as indicated. *E)* Co-IP assays to show that the TIP-1 presence enhanced the LZAP interaction with ARF within the irradiated D54 cells, whereas depletion of TIP-1 reduced the complex formation between LZAP and ARF within the irradiation glioma cells. Shown are representative images of at least two independent experiments.

To determine the mechanism by which TIP-1 modulates the p53 protein ubiquitination, a yeast-two-hybrid screening was conducted to identify TIP-1-interacting proteins. Among the hits from the screening (Fig. *7A*), we have particularly focused on LZAP which contains a typical PDZ-binding motif at its C-terminal end (Fig. *7B*) and had been characterized as a tumor suppressor that regulates tumor cell response to multiple genotoxic stresses including IR through binding ARF and modulating the HMD-2 mediated p53 ubiquitination and activation [12], [13], [34]. To validate the protein interaction between TIP-1 and LZAP, *in vitro* pull-down assays were conducted with recombinant GST-tagged TIP-1 proteins that were purified to homogeneity [25]. As shown in Figure *7C*, the endogenous LZAP protein was only precipitated with the wild type (WT) of TIP-1 from D54 cell lysates. Neither GST control nor one TIP-1 mutant (MUT) that contains a dysfunctional PDZ domain (Fig. *7B*) bound LZAP protein in the pull-down assay. To study the i*n vivo* protein interaction, a LZAP mutant was created by replacing critical amino acids in the putative C-terminal PDZ-binding motif with alanine (Fig. *7B*, from –GTSL to -GASA). The mutation was verified with DNA sequencing. The FLAG-tagged LZAP, wild type (WT) or mutant (MUT), was used to co-transfect D54 cells with WT or MUT of c-myc-tagged TIP-1 (Fig. *7D*), respectively. The *in vivo* protein interaction was studied with co-IP of the protein complex from cell lysates followed by SDS-PAGE fractionation and immunoblot with specific antibodies. The results (Fig. *7D*) indicated that the protein interaction between TIP-1 and LZAP is mediated by the PDZ domain within TIP-1 and the C-terminal PDZ-binding motif of LZAP. Mutation at either part destroyed the specific protein interaction.

### TIP-1 enhances the protein interaction between LZAP and ARF in the irradiated glioma cells

LZAP was reported as one ARF-interacting protein that modulates HDM2-mediated p53 protein ubiquitination and activation [12]. Co-immunoprecipitation revealed that an ectopic TIP-1 expression enhanced the physical interaction between LZAP and ARF, while TIP-1 knockdown attenuated the association of the two proteins in the irradiated D54 cells (Fig. *7E*). In the untreated cells, only a moderate impact of the presence of TIP-1 protein on the LZAP interaction with ARF was observed. It was also noted that, under the experimental conditions, none of TIP-1, LZAP or ARF was significantly affected by X-ray irradiation at the protein level (Supplementary Figs. *S1A, B* and unpublished data).

### LZAP mediates the TIP-1-regulated p53 polyubiquitination

**Figure 8:**
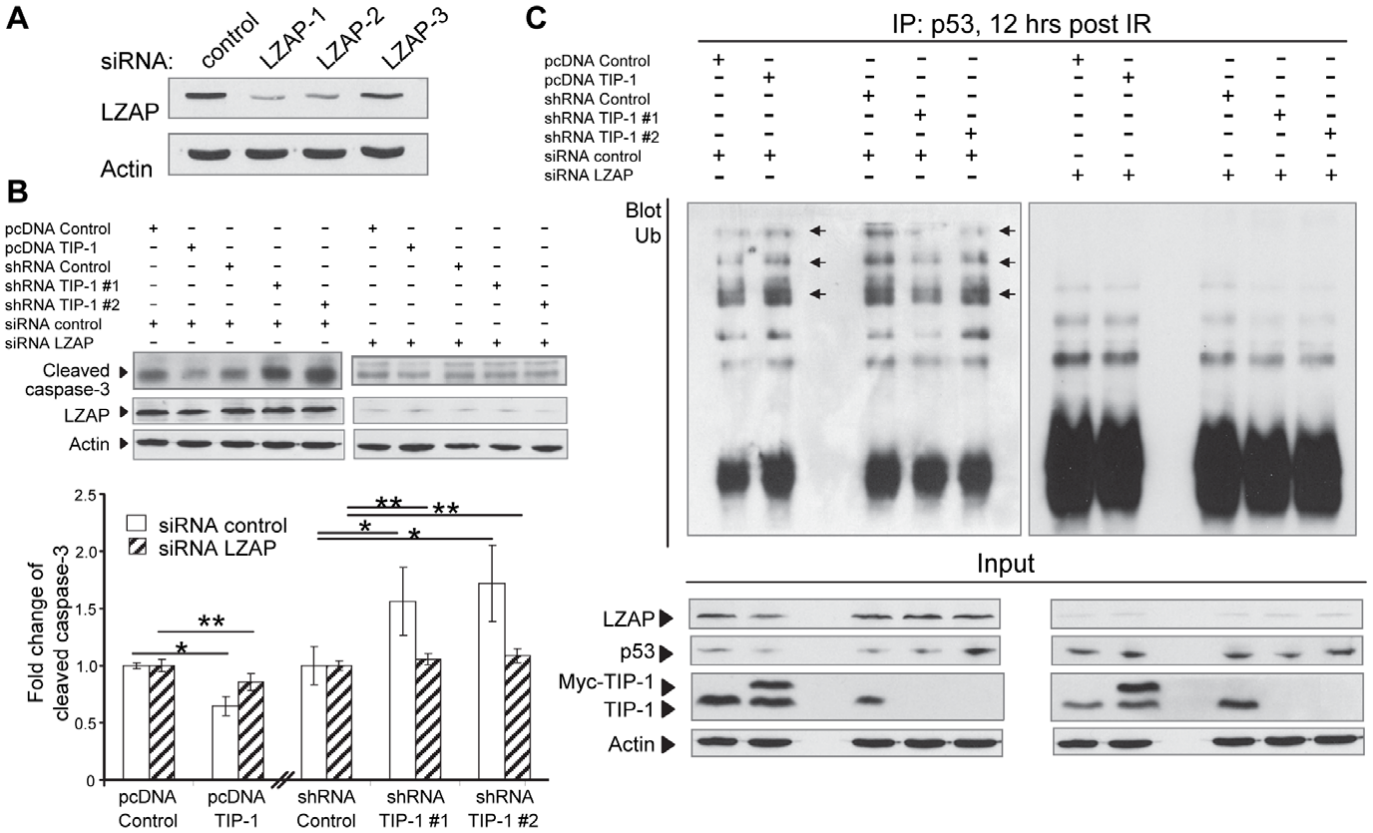
LZAP is required for the TIP-1-regulated IR-induced apoptosis and p53 polyubiquitination in D54 glioma cells. *A)* Western blot analysis of LZAP knockdown in D54 glioma cells with siRNAs. Whole cell lysates were analyzed at 72 hours post the transfection. A validated control siRNA was included. *B)* Western blot analysis and quantification of caspase-3 cleavage with or without LZAP knockdown. Cells were transfected with LZAP-targeting siRNA or a control siRNA 72 hours prior to irradiation at 5 Gy. Whole cell lysates were analyzed at 24 hours post IR treatment. Significance was determined by comparing the denoted group to the respective vector control. *p<0.05; ** *p*>0.05 with t-test. *C)* Western blot analysis of the p53 protein levels and ubiquitination with or without LZAP knockdown. The cells were irradiated at 5 Gy. MG132 was added at 8 hours post IR treatment and incubated for 4 more hours before the cell lysates were collected to analyze the p53 ubiquitination. The representative image shows the ubquitinated p53 ladder (pointed with arrows) as well as overall LZAP, p53, TIP-1 and actin in each cell lysate.

To determine roles of the protein interaction between TIP-1 and LZAP in the TIP-1-regulated radioresistance of glioma cells, especially the p53 protein polyubiquitination or accumulation after IR, a panel of siRNAs targeting three independent regions of LZAP gene transcripts was tested in the D54 cells (Fig. *8A*). One siRNA (LZAP-1) that most efficiently down regulated the LZAP expression was selected for the rest experiments. The LZAP siRNA was introduced to the D54 cells with variable TIP-1 expression levels. The cells were treated with or without IR (5 Gy) before apoptosis (caspase-3 activation) and p53 ubiquitination assays. Western blot analyses indicated that depleting LZAP with siRNA attenuated the effects of TIP-1 expression on the IR-induced D54 cell apoptosis as indicated with caspase-3 cleavage assays (Fig. *8B*). Analyses of the overall p53 protein levels and polyubiquitination status (Fig. *8C*) indicated that the impact of TIP-1 expression on the p53 protein accumulation and polyubiquitination within the irradiated glioma cells were remarkably reduced in the absence of LZAP protein.

### TIP-1 knockdown sensitizes D54 xenografts to X-ray radiation *in vivo*

**Figure 9:**
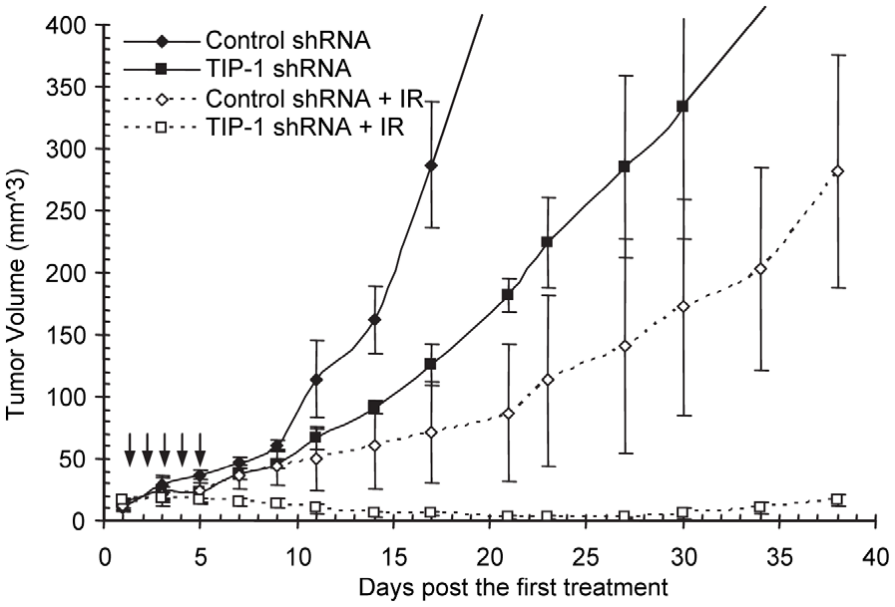
TIP-1 depletion sensitized D54 tumor xenografts to IR *in vivo*. Stable transfected D54 cells with a control or TIP-1 targeting shRNA were implanted subcutaneously in hind limbs of Foxn1-null nude mice. Tumor sizes were measured with caliper, and irradiation (3 Gy for five consecutive days as indicated with arrows) was delivered to tumors when the tumor size reached 3 mm in diameter. *n* = 9 in each group.

A tumor growth study was conducted to test the feasibility of antagonizing TIP-1 for an improved radiotherapy of malignant glioma by using a mouse model bearing subcutaneous human glioma xenografts. Stable transfected D54 cells with a control shRNA or TIP-1-specific shRNA were subcutaneously implanted in FoxN1-null nude mice. It was found that TIP-1 depletion retarded the tumor growth in nude mice (Fig. 9 and unpublished data). To determine the impact of the TIP-1 expression status on the radiation response of tumor in vivo, fractions of X-ray radiation (3 Gy for five consecutive days) was delivered when the tumors reached same size (around 3 mm in diameter). In the treatment course, only the tumors were irradiated while the other parts of mouse body were shielded. Tumor size was measured with caliper in and beyond the treatment courses. Compared to the tumors with a control shRNA that regrew after the treatment course (Fig. 9), the D54 xenografts with a TIP-1-specific shRNA did not assume tumor growth after the IR treatment course. Indeed, tumors eventually shrunk and some were undetectable by day 30 post the IR treatment.

## Discussion

**Figure 10:**
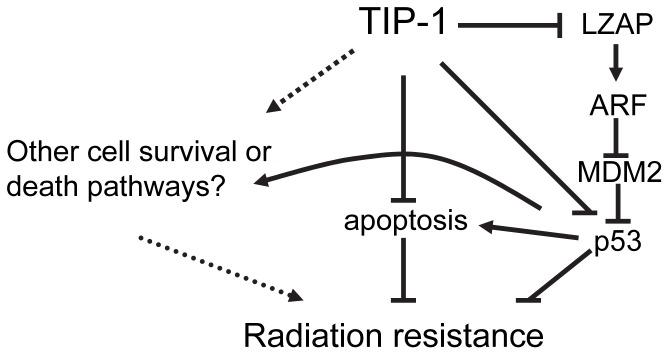
Schemetic illustration of the putative roles of TIP-1 in the radioresistance of malignant gliomas.

The majority of cancer patients have received radiotherapy in their tour to fight against cancers. Unfortunately, the therapeutic efficacy of radiation is largely limited by intrinsic or acquired resistance in patients with advanced malignant gliomas (grade III or IV). A significant portion of cancer research is to identify the genetic or epigenetic factors that contribute to the radioresistance and eventually could be targeted to improve the therapeutic efficacy of radiotherapy of cancer. This study has identified TIP-1 as one novel protein that confers the radioresistance in malignant glioma cells. Studies with established glioma cell lines demonstrated that expression of TIP-1 conferred resistance of glioma cells to X-ray radiation while TIP-1 knockdown sensitized glioma cells to IR. Biochemical studies further identified that the TIP-1 regulates the radioresistance of glioma cells, at least in part, through promoting p53 protein polyubiquitination for degradation. LZAP was identified as one novel TIP-1-interacting protein in this study, the presence of TIP-1 modulated the functionality of LZAP towards p53 polyubiquitination and degradation. Finally, in a mouse model with subcutaneous xenografts of human malignant glioma, it was demonstrateed that depleting TIP-1 within glioma cells inhibited the tumor regrowth after IR treatment. Data provided in this study support one conclusion that TIP-1 is a novel regulator in the radioresistance of malignant gliomas. The putative roles of TIP-1 in the radiation resistance were summarized in Fig. *10*. Considering the high TIP-1 expression levels in the advanced human malignant gliomas, this study suggested that antagonizing TIP-1 might represent one novel approach for an improved radiotherapy of the advanced malignant gliomas.

p53 plays a central role in determining the cell fate after genotoxic stress by mediating the DNA-damage-induced cell cycle arrest, senescence or apoptosis. p53 is a short-lived protein that is normally expressed at low level owing to its interaction with HDM2. HDM2 functions as an E3 ubiquitin ligase and promotes the proteasome-mediated p53 protein degradation [5]. DNA damage triggers p53 phosphorylation that blocks the interaction of p53 with MDM2 leading to the stabilization and activation of p53 [35]. Activation of p53 is also regulated by ARF which releases p53 from the HDM2-mediated ubiquitination, or sequesters the HDM2-p53 complex in the cellular nucleus and prevents p53 degradation [6], [36]. The complexity of p53 regulation was further demonstrated with discovery of up- and down-stream modulators. For example, in a human osteosarcoma cell line (U2OS), LZAP formed a ternary protein complex with ARF and HMDM2, and reversed the inhibitory ability of ARF on the HDM2's ubiquitin ligase activity towards p53 degradation. Indeed, LZAP co-operated with ARF in maintaining p53 stability and increasing the p53 transcriptional activity probably through promoting mono-ubiquitination and nuclear translocation of p53 protein [12]. Opposite to those observations, this study provided evidences showing that TIP-1 is a novel interacting protein of LZAP in glioma cells, the presence of TIP-1 enhanced the protein interaction between LZAP and ARF, promoted the polyubiquitination of p53 and resulted in a reduced p53 protein accumulation after irradiation. We did not detect a significant difference regarding the ternary complex formed with LZAP, ARF and HMD2 or the subcellular distribution of p53 at variable TIP-1 expression levels (data not shown). Those data might in part reflect the complexity of p53 regulation and influence of cellular context in different type of tumors, but also suggest a new mechanism of action (MOA) of LZAP in human malignant glioma, that is, complex formation between LZAP and ARF might modulate the functionality of HMD2 towards the p53 polyubiquitination and proteasome-mediated degradation. Regarding the heterogeneous roles of LZAP in cancer biology, it was noted that a controversial function of LZAP was recently reported in human hepatocellular carcinomas [14].

TIP-1 has demonstrated a variety of biological functions, such as modulating the cell proliferation, migration and polarization through selective interaction with β-catenin [17], Rhotekin [20], glutaminase L [37], potassium channel Kir 2.3 [38] and others [19], [21], [22], respectively. Although several studies have connected TIP-1 with cell transformation and oncogenesis [18], [19], [25], [39], yet its biological functions and the associated mechanisms remain largely elusive. This study provided the first ever evidences showing that TIP-1 is involved in the radioresistance of malignant gliomas. A time-course study showed that glioma cells with TIP-1 expression were more efficient to repair the IR-induced DNA damage, while TIP-1 knockdown delayed DNA repair in the irradiated glioma cells. The delayed DNA damage repair after IR was translated into an increased apoptosis and a reduced survival fraction of the glioma cells with TIP-1 depletion. Interestingly, a significant amount of DNA damage were detected with comet assay and γ-H2Ax foci staining (Fig. *4*) in the TIP-1-depleted glioma cells even without IR treatment, suggesting that TIP-1 might plays a vital role in the genome stability or integrity of glioma cells. It was also noted that the impact of the TIP-1 expression status on apoptosis of the glioma cell was not as dramatic as that observed in clonogenic assays, suggesting more studies are needed to explore the putative roles of pathways other than apoptosis, such as senescence or auophagy [35], in the TIP-1 regulated glioma cell response to IR.

Restoration of p53 functionality has been tested in preclinical and clinical settings as one approach to treat tumors. However, the outcomes are contrary due to the complexity of the p53 activation and its functionality in a diversified background of tumors [7], [9], [40]. Even though a significant portion of the primary malignant gliomas express the wild-type p53 protein, the status of p53 expression has a poor prognostic value in predicting the therapeutic outcomes of the primary malignant gliomas [10], [11]. This study revealed a novel mechanism of p53 regulation in malignant glioma cells. *In vitro* studies indicated that the overexpressed TIP-1 in glioma cells inhibited p53 activation after irradiation, depleting TIP-1 resulted in an elevated accumulation of p53 protein and improved the cytotoxicity of IR. Studies with tumor xenografts showed that depleting TIP-1 inhibited the tumor regrowth after IR treatment, leading to tumor shrink or even disappearance in some animals. These data suggest that antagonizing TIP-1 might represent one novel approach to improve the p53-targeted therapies of malignant gliomas.

## Supporting Information

Figure S1 Impact of X-ray irradiation on the TIP-1 expression levels in D54 cells. *(A)* Monolayer of D54 cells were irradiated with variable doses of X-ray and cell lysates were prepared for SDS-PAGE and western blot profiling of protein expression at 12 hours post the irradiation. Mock (0 Gy) treated cells were used as control. (B) Monolayer of D54 cells were irradiated with 5 Gy of X-ray and cell lysates were prepared for SDS-PAGE and western blot profiling of protein expression at variable time points post the irradiation. Mock (0 Gy) treated cells were used as control. Actin was blotted in each sample.
(PDF)
