# Supplementary figures and images for: Expression of TIP-1 Confers Radioresistance of Malignant Glioma Cells
Source: PLoS One. 2012 Sep 17;7(9):e45402. doi: 10.1371/journal.pone.0045402 (PMC3444456; doi:10.1371/journal.pone.0045402)

A

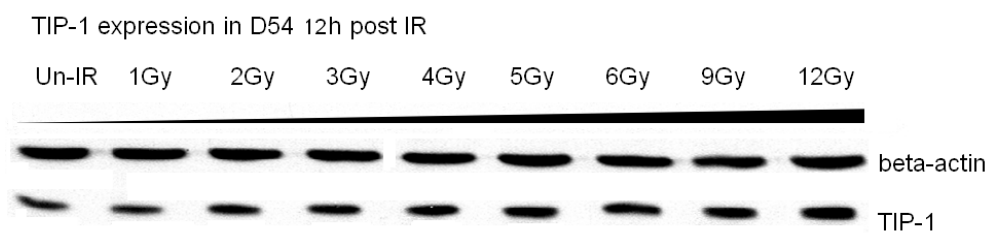

B

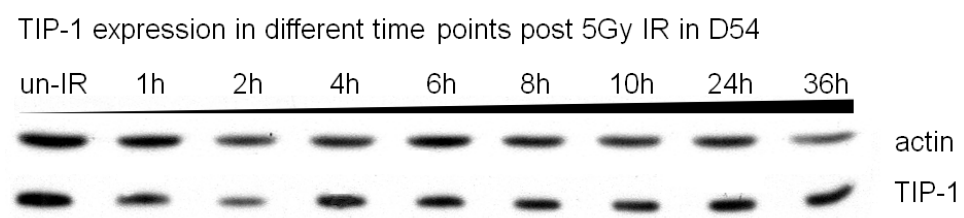

Supplement: Figure S1 — Impact of X-ray irradiation on the TIP-1 expression levels in D54 cells. (A) Monolayer of D54 cells were irradiated with variable doses of X-ray and cell lysates were prepared for SDS-PAGE and western blot profiling of protein expression at 12 hours post the irradiation. Mock (0 Gy) treated cells were used as control. (B) Monolayer of D54 cells were irradiated with 5 Gy of X-ray and cell lysates were prepared for SDS-PAGE and western blot profiling of protein expression at variable time points post the irradiation. Mock (0 Gy) treated cells were used as control. Actin was blotted in each sample. (PDF) [file pone.0045402.s001.pdf]
